# Supplementary material for: Sex-biased gene expression is repeatedly masculinized in asexual females
Source: Nat Commun. 2019 Oct 11;10:4638. doi: 10.1038/s41467-019-12659-8 (PMC6789136; doi:10.1038/s41467-019-12659-8)
Supplement: Supplementary file 1 — Supplementary Information [file 41467_2019_12659_MOESM1_ESM.pdf]

Supplementary Information

Sex-biased gene expression is repeatedly masculinized in asexual females

Parker et al.

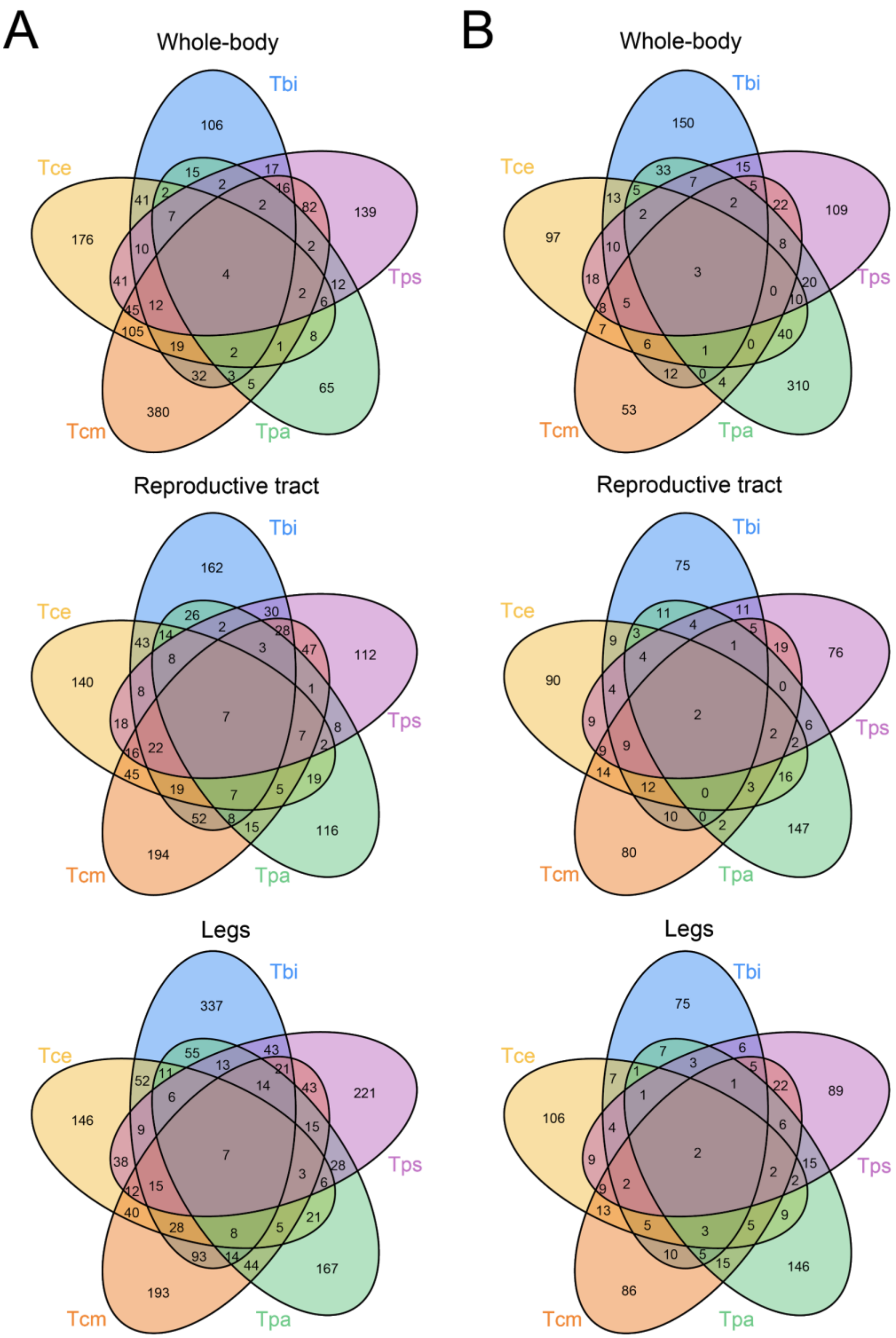

Supplementary Figure 1 | Venn-diagrams showing the overlap of enriched GO-terms for (**A**) female-biased and (**B**) male-biased genes. Species names are given as abbreviations: Tbi = *T. bartmani*, Tce = *T.cristinae*, Tps = *T. poppensis*, Tcm = *T. californicum*, Tpa = *T. podura*).

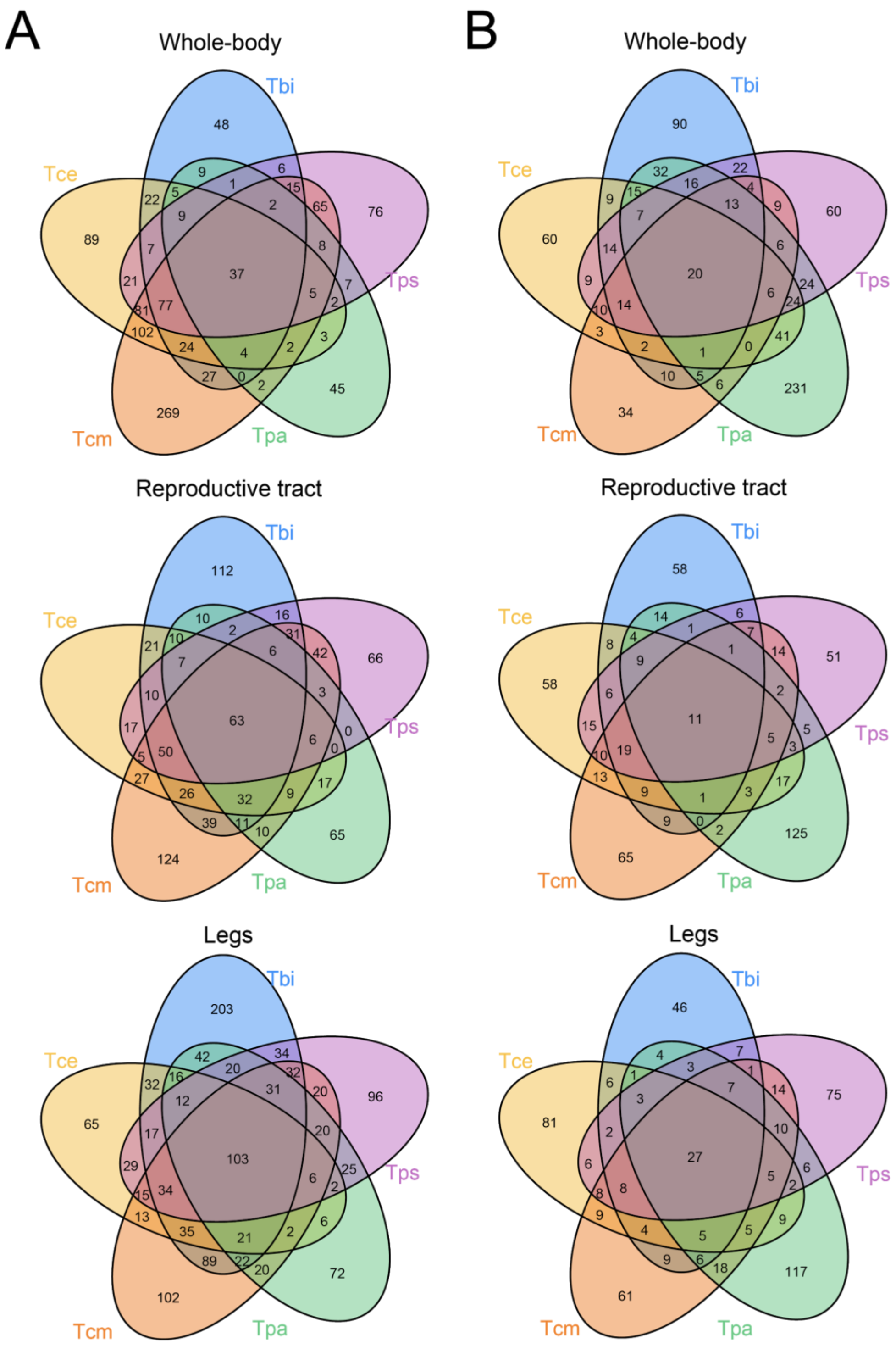

Supplementary Figure 2 | Venn-diagrams showing the overlap of enriched GO-terms for (**A**) female-biased and (**B**) male-biased genes when GO terms were first clustered together based on parent or child terms. Species names are given as abbreviations: Tbi = *T. bartmani*, Tce = *T. cristinae*, Tps = *T. poppensis*, Tcm = *T. californicum*, Tpa = *T. podura*).

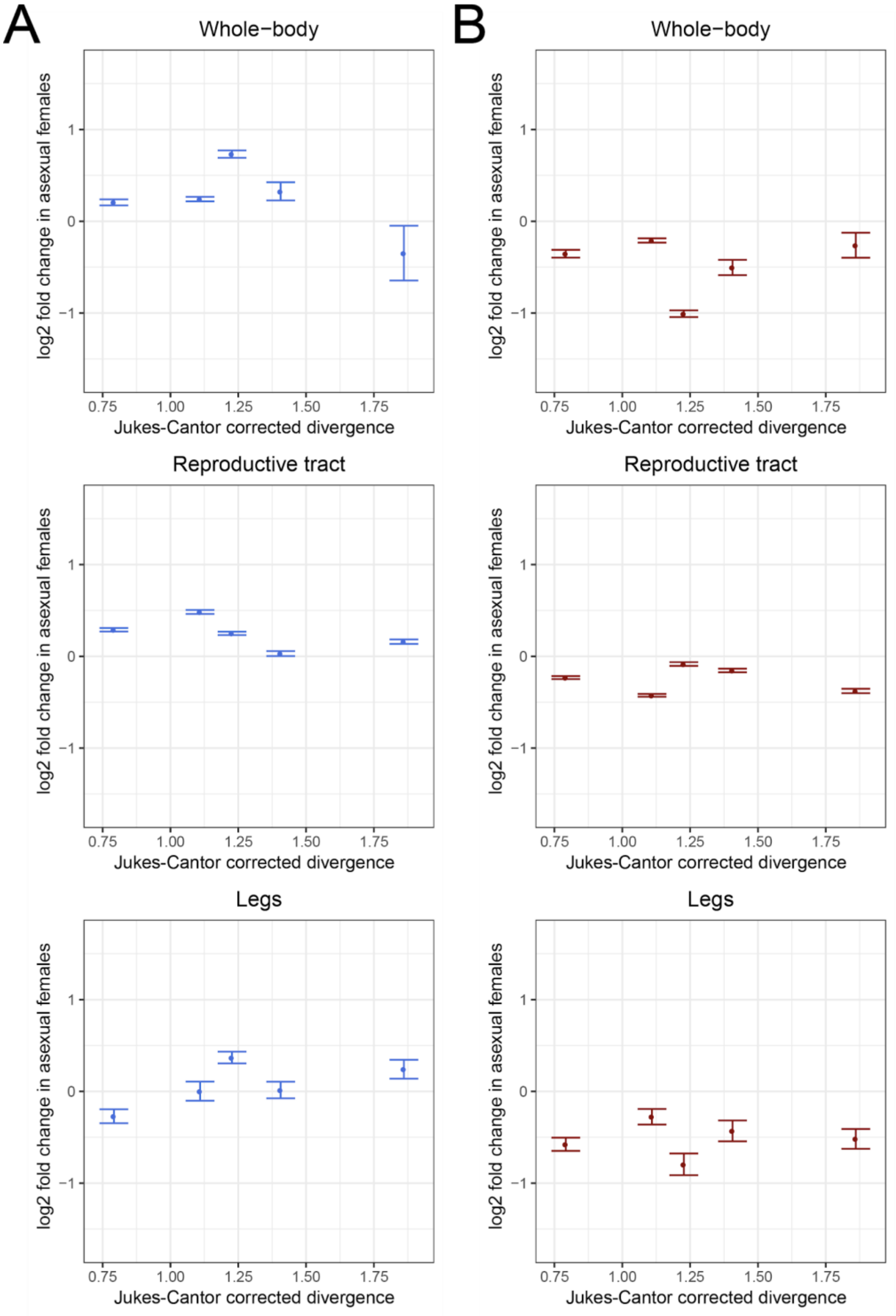

Supplementary Figure 3 | Relationship between the shift in male-biased (A) or female-biased (B) gene expression in asexual females and sexual-asexual divergence time (estimated as from Jukes–Cantor corrected divergence from Bast et al. [1]) in whole-bodies, reproductive tracts, and

legs. Each point is the mean shift in expression for an asexual species with standard error indicated by the error bars. Source data are provided as a Source Data file.

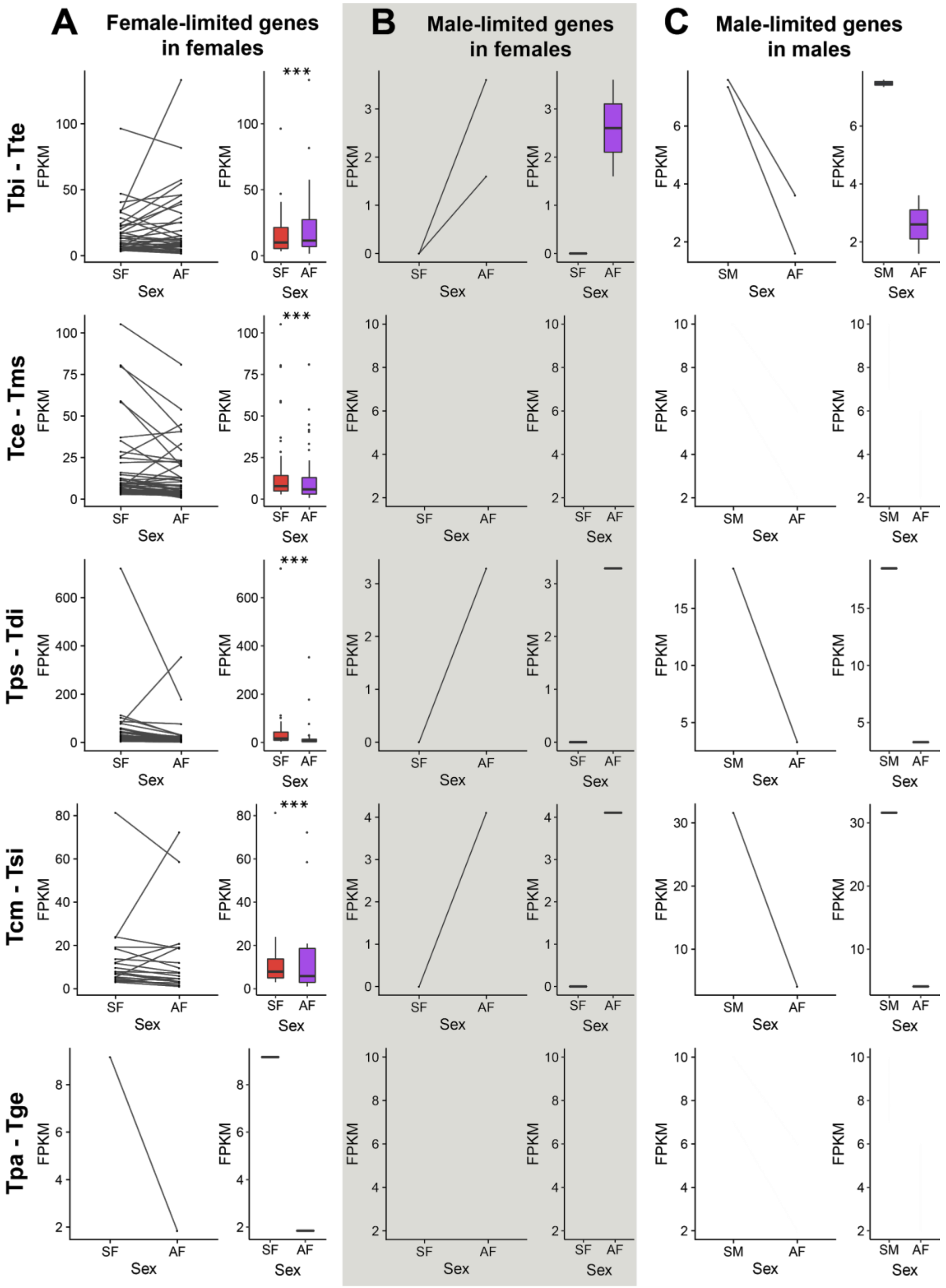

Supplementary Figure 4 | Expression of sex-limited genes in the whole-body. A) Expression of female-limited genes in sexual females (SF, red) and asexual females (AF, purple), B) Expression of male-limited genes in sexual females (SF, red) and asexual females

(AF, purple), C) Expression of male-limited genes in sexual males (SM, blue) and asexual females (AF, purple). Asterisks indicate the significance level (FDR) of Wilcoxon tests (*** < 0.001, ** < 0.01, * < 0.05). Species names are given as abbreviations in the form sexual-species - asexual species at the left-hand side (Tbi = *T. bartmani*, Tce = *T.cristinae*, Tps = *T. poppensis*, Tcm = *T. californicum*, Tpa = *T. podura*, Tte = *T. tahoe*, Tms = *T. monikensis*, Tdi = *T. douglasi*, Tsi = *T. shepardi*, and Tge = *T. genevievae*). For the boxplots, boxes represent the interquartile range (25th and 75th percentiles) of the data with the line inside the box representing the median. Whiskers show the most extreme value in the data which is no more than 1.5 times the interquartile range from the box. Source data are provided as a Source Data file.

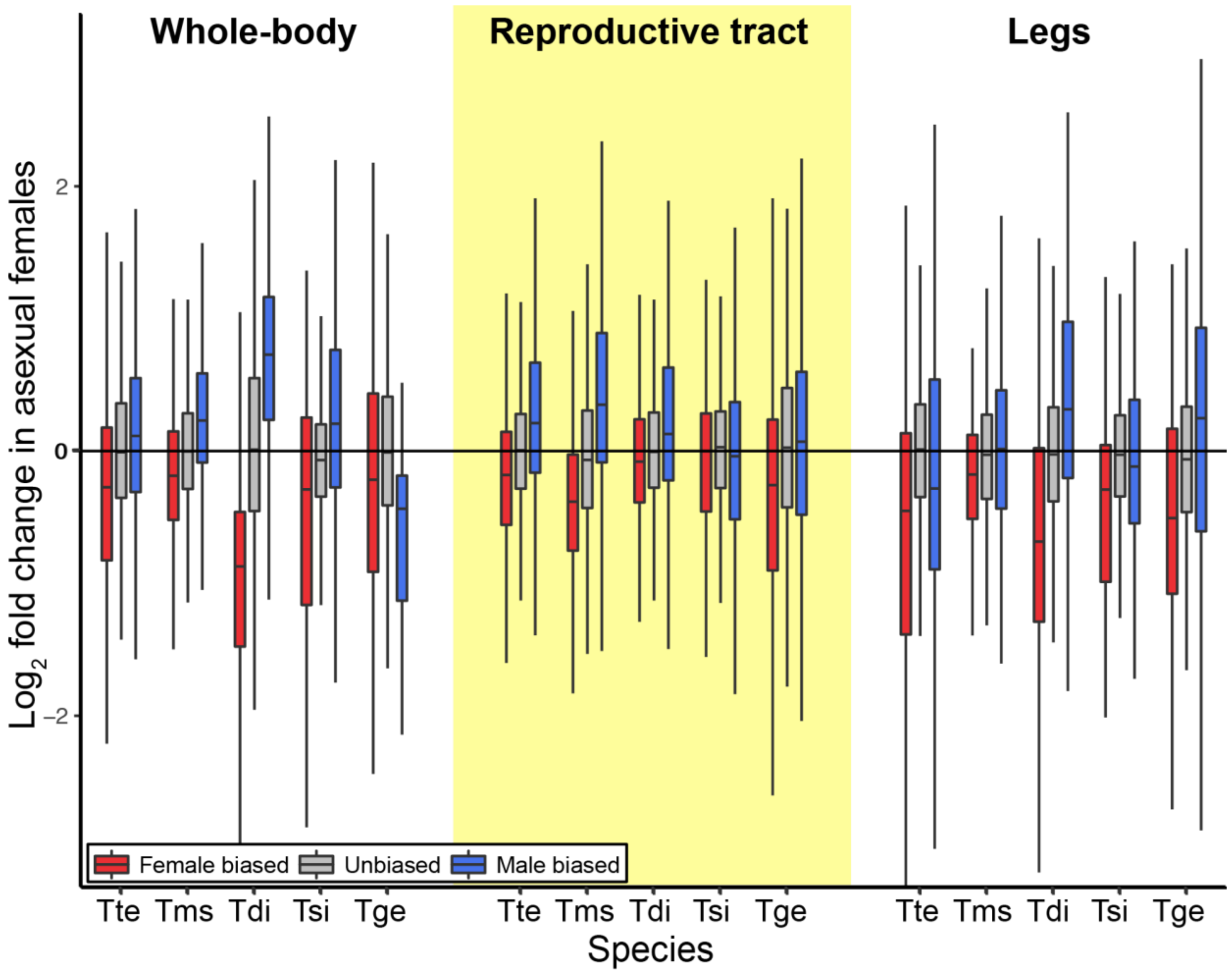

Supplementary Figure 5 | Expression shift in sex-biased genes in asexual females when including genes with low expression in asexual females. Positive values on the y-axis indicate increased expression in asexual females. Asterisks indicate the significance level (FDR) of Wilcoxon tests comparing the change in expression in female-biased (red) and male-biased (blue) genes to unbiased genes (*** < 0.001, ** < 0.01, * < 0.05). Species names are abbreviated as follows: Tte = *T. tahoe*, Tms = *T. monikensis*, Tdi = *T. douglasi*, Tsi = *T. shepardi*, and Tge = *T. genevievae*. Boxes represent the interquartile range (25th and 75th percentiles) of the data with the line inside the box representing the median. Whiskers show the most extreme value in the data which is no more than 1.5 times the interquartile range from the box. Source data are provided as a Source Data file.

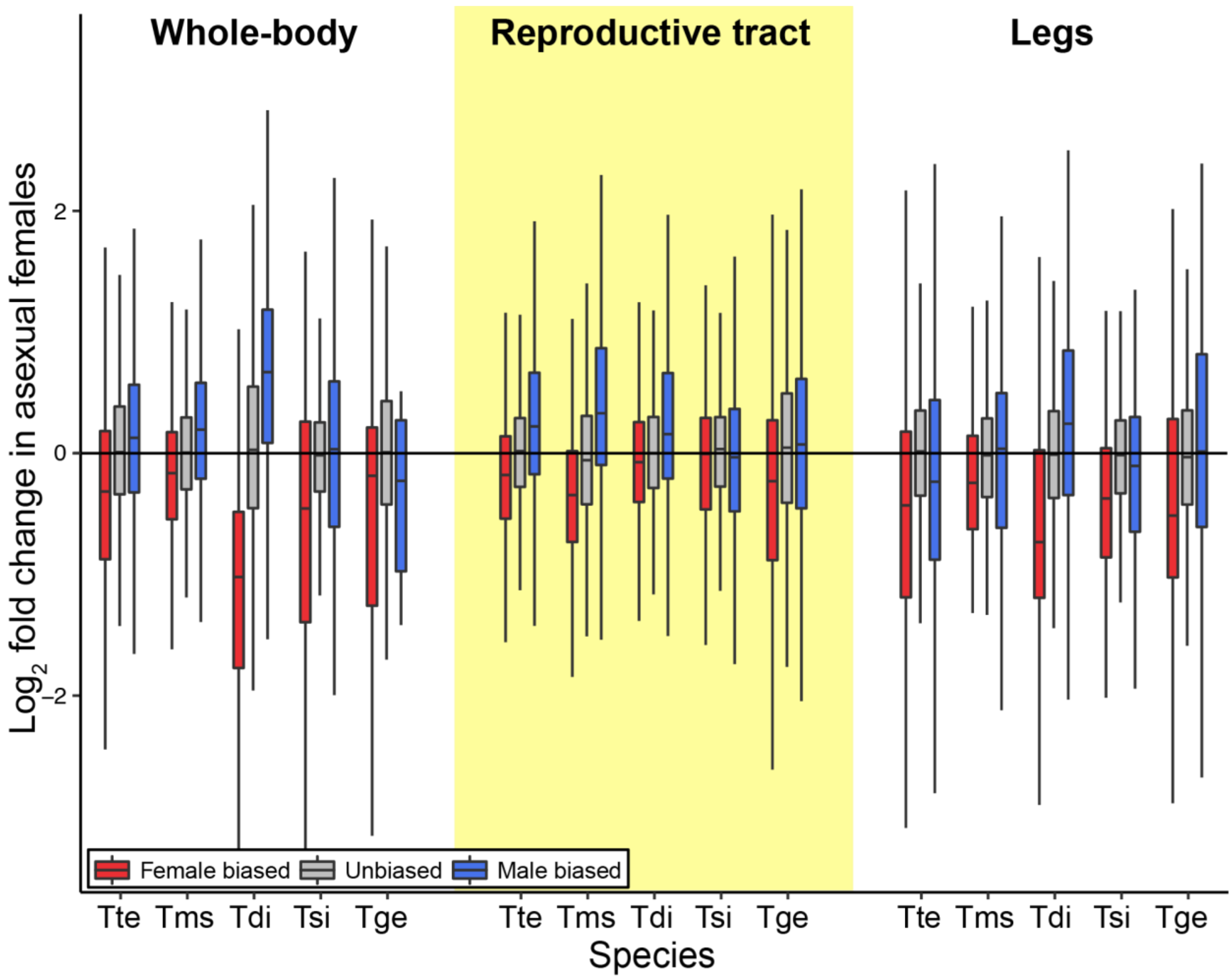

Supplementary Figure 6 | Expression shift in sex-biased genes in asexual females when using the full sexual-sister species transcriptome as a reference. Positive values on the y-axis indicate increased expression in asexual females. Species names are abbreviated as follows: Tte = *T. tahoe*, Tms = *T. monikensis*, Tdi = *T. douglasi*, Tsi = *T. shepardi*, and Tge = *T. genevievae*. Boxes represent the interquartile range (25th and 75th percentiles) of the data with the line inside the box representing the median. Whiskers show the most extreme value in the data which is no more than 1.5 times the interquartile range from the box. Source data are provided as a Source Data file.

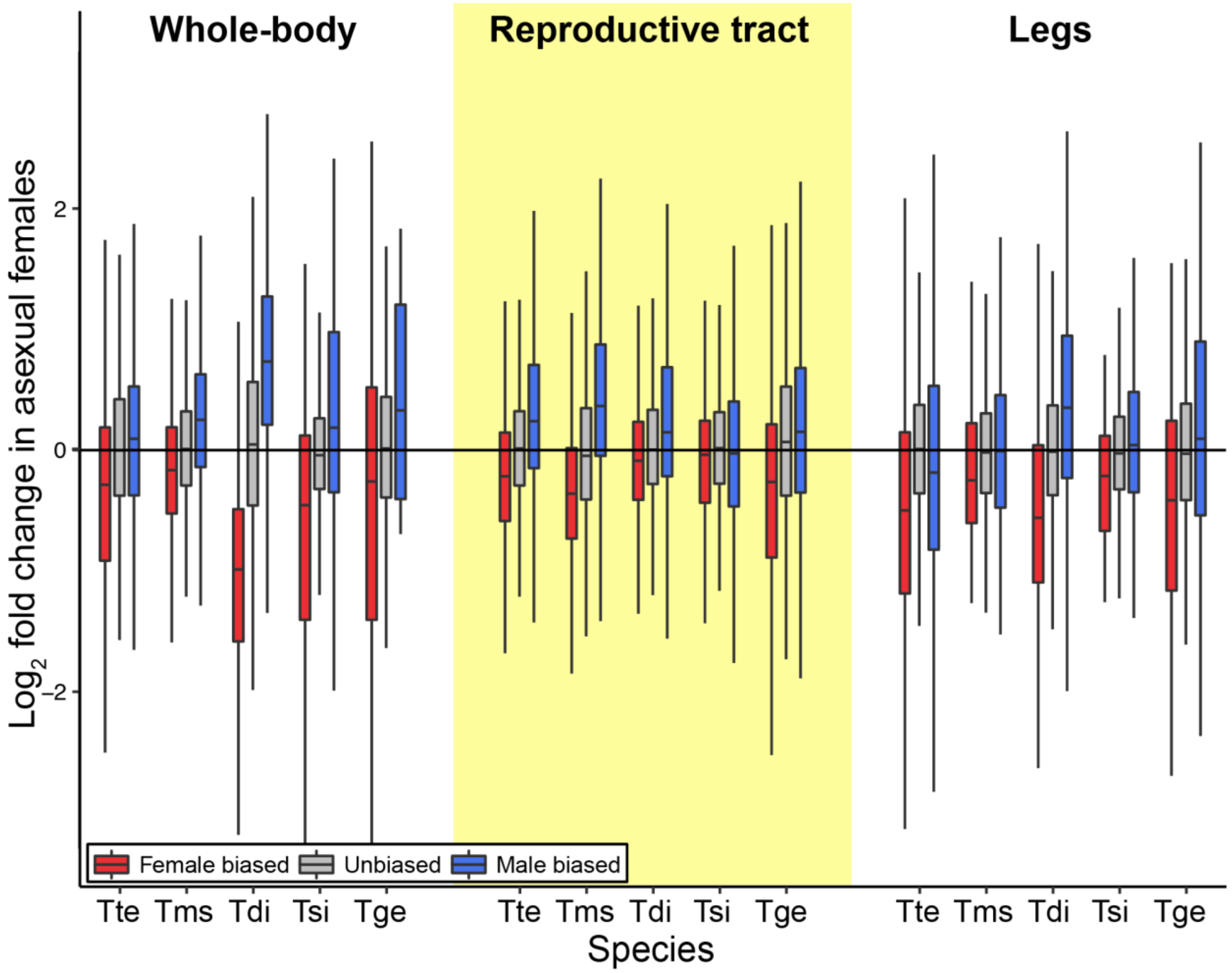

Supplementary Figure 7 | Expression shift in sex-biased genes in asexual females when using the full asexual-sister species transcriptome as a reference. Positive values on the y-axis indicate increased expression in asexual females. Species names are abbreviated as follows: Tte = *T. tahoe*, Tms = *T. monikensis*, Tdi = *T. douglasi*, Tsi = *T. shepardi*, and Tge = *T. genevievae*. Boxes represent the interquartile range (25th and 75th percentiles) of the data with the line inside the box representing the median. Whiskers show the most extreme value in the data which is no more than 1.5 times the interquartile range from the box. Source data are provided as a Source Data file.

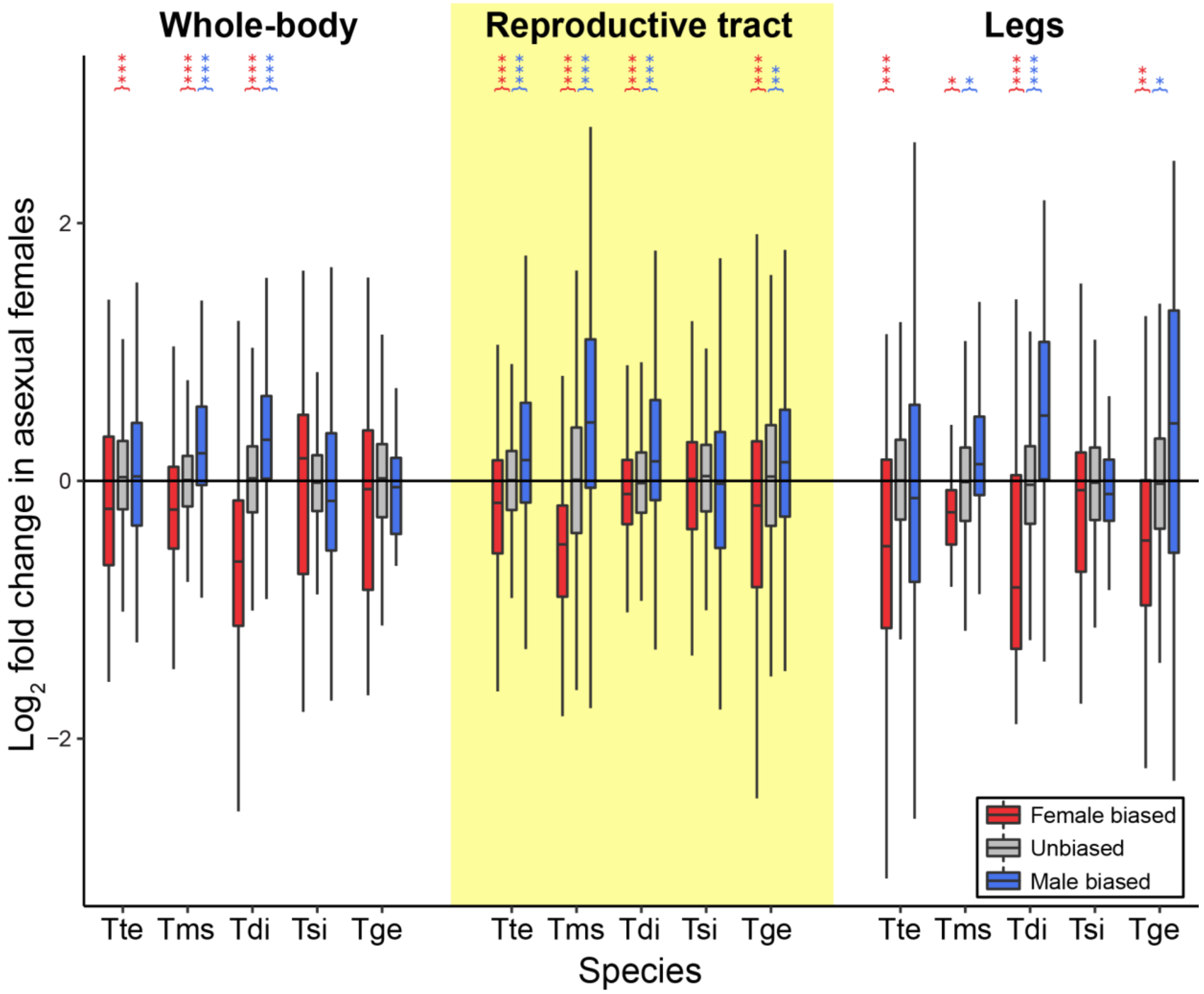

Supplementary Figure 8 | Expression shift in sex-biased genes in asexual females when using the 10-species orthologs only. Positive values on the y-axis indicate increased expression in asexual females, negative values indicate decreased expression. Asterisks indicate the significance level (FDR) of Wilcoxon tests comparing the change in expression in female-biased (red) and male-biased (blue) genes to unbiased genes (*** < 0.001, ** < 0.01, * < 0.05). Species names are abbreviated as follows: Tte = *T. tahoe*, Tms = *T. monikensis*, Tdi = *T. douglasi*, Tsi = *T.shepardi*, and Tge = *T. genevievae*. Boxes represent the interquartile range (25th and 75th percentiles) of the data with the line inside the box representing the median. Whiskers show the most extreme value in the data which is no more than 1.5 times the interquartile range from the box. Source data are provided as a Source Data file.

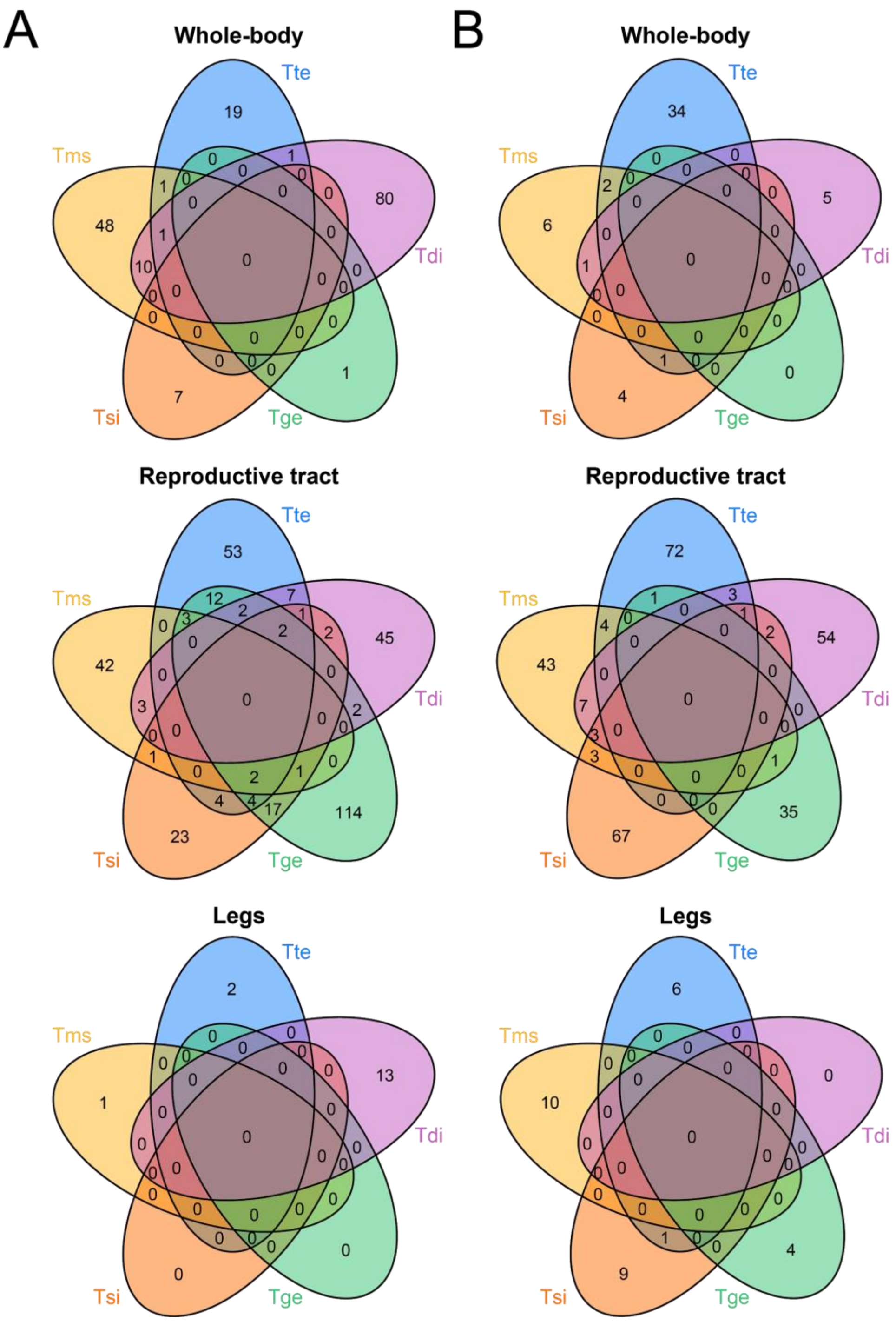

Supplementary Figure 9 | Overlap of GO-terms enriched in A) female-biased genes with decreased expression in asexual females, and B) male-biased genes with increased expression in asexual females. Species names are abbreviated as follows: Tte = *T. tahoe*, Tms = *T. monikensis*, Tdi = *T. douglasi*, Tsi = *T.shepardi*, and Tge = *T. genevievae*.

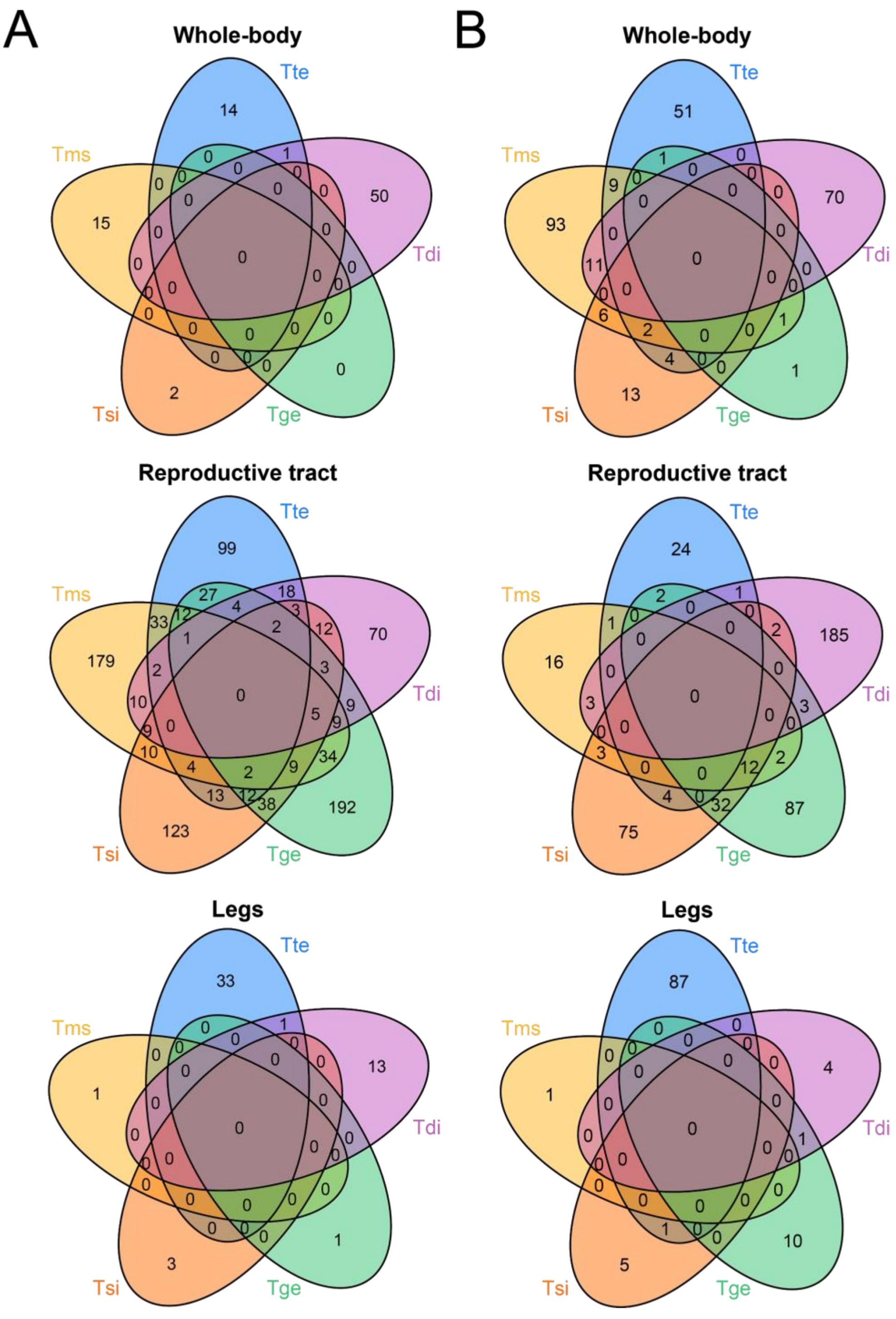

Supplementary Figure 10 | Overlap of GO-terms enriched in A) female-biased genes with increased expression in asexual females, and B) male-biased genes with decreased expression in asexual females. Species names are abbreviated as follows: Tte = *T. tahoe*, Tms = *T. monikensis*, Tdi = *T. douglasi*, Tsi = *T.shepardi*, and Tge = *T. genevievae*.

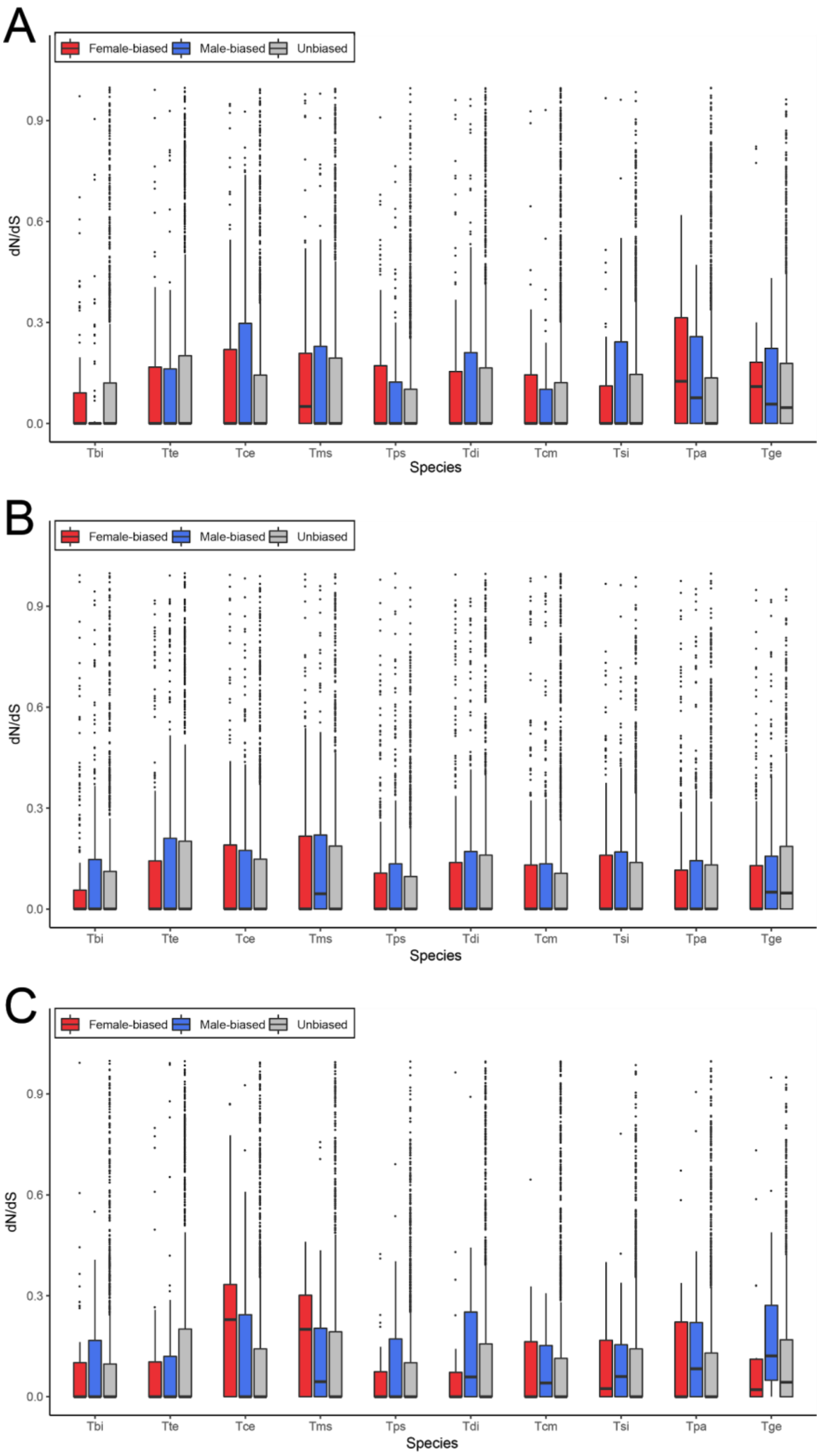

Supplementary Figure 11 | dN/dS ratios for female-biased (red), male-biased (blue) and unbiased (grey) genes in each of the species. Species names are abbreviated as follows: Tbi

= *T. bartmani*, Tce = *T. cristinae*, Tps = *T. poppensis*, Tcm = *T. californicum*, Tpa = *T. podura*, Tte = *T. tahoe*, Tms = *T. monikensis*, Tdi = *T. douglasi*, Tsi = *T. shepardi*, and Tge = *T. genevievae.* sexual-asexual sister species pairs are as follows: Tbi-Tte, Tce-Tms, Tps-Tdi, Tcm-Tsi, Tpa-Tge. Boxes represent the interquartile range (25th and 75th percentiles) of the data with the line inside the box representing the median. Whiskers show the most extreme value in the data which is no more than 1.5 times the interquartile range from the box. Source data are provided as a Source Data file.

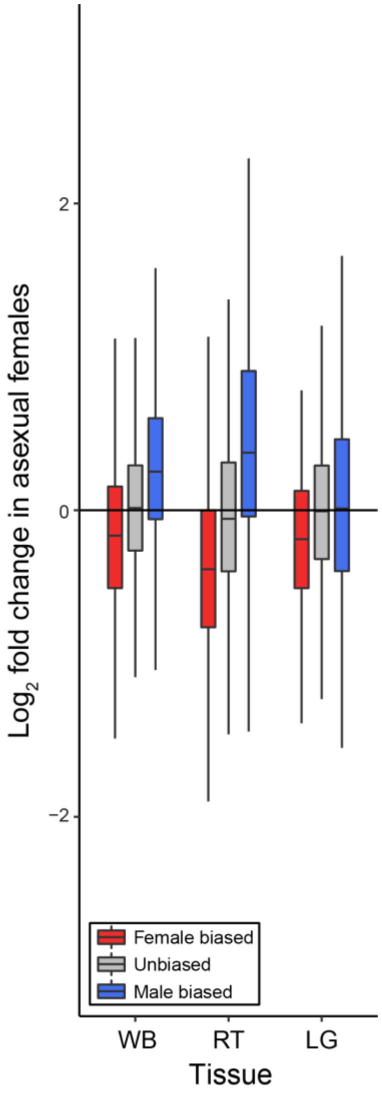

Supplementary Figure 12 | Expression shift in autosomal sex-biased genes in asexual female *T. monikensis* in whole-bodies (WB), reproductive tracts (RT) and legs (LG). Boxes represent the interquartile range (25th and 75th percentiles) of the data with the line inside the box representing the median. Whiskers show the most extreme value in the data which is no more than 1.5 times the interquartile range from the box. Source data are provided as a Source Data file.

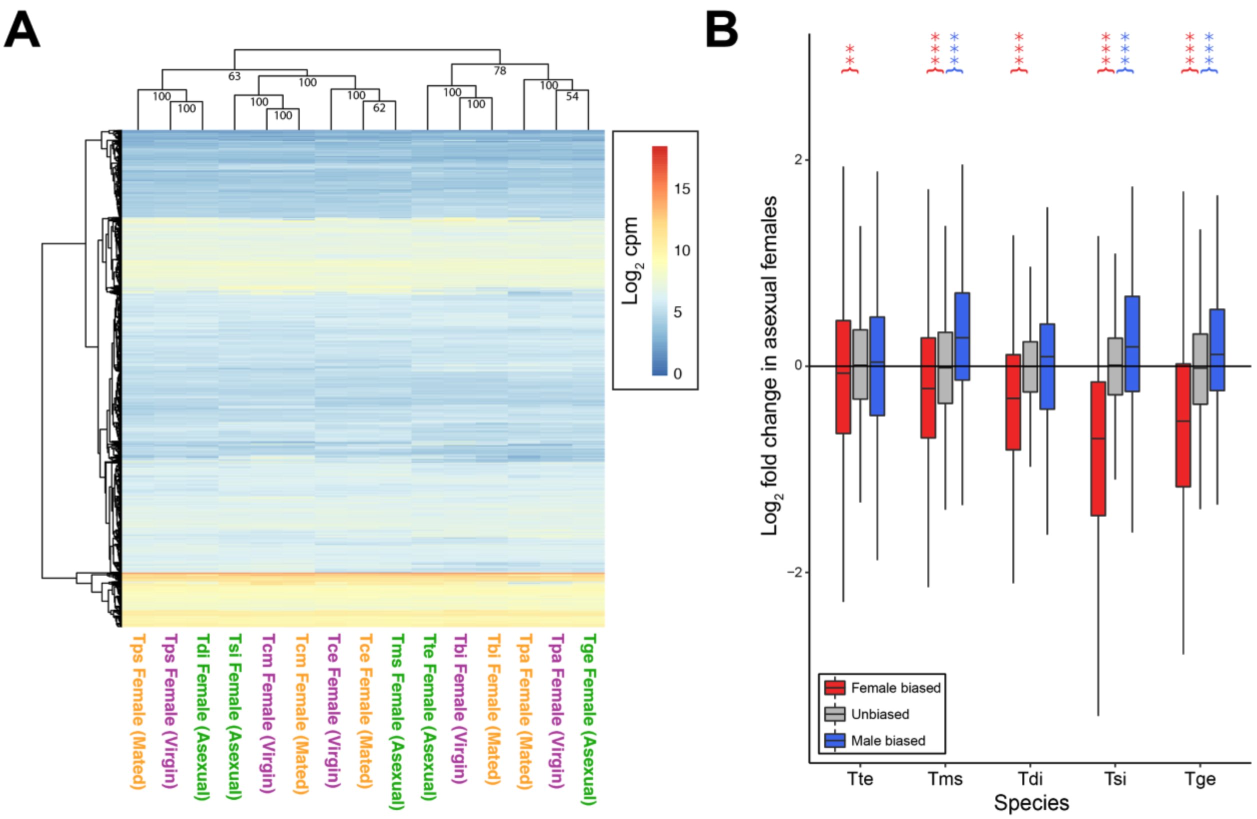

Supplementary Figure 13 | **A.** Heatmap of expression in whole-bodies shows that whether virgin sexual females have a more similar expression to mated sexual females or asexual females depends upon species. Values on each node show the bootstrap support from 10,000 replicates. **B** Expression shift in sex-biased genes in asexual females in whole-bodies when using virgin sexual female samples. Species names are abbreviated as follows: Tbi = *T. bartmani*, Tce = *T. cristinae*, Tps = *T. poppensis*, Tcm = *T. californicum*, Tpa = *T. podura*, Tte = *T. tahoe*, Tms = *T. monikensis*, Tdi = *T. douglasi*, Tsi = *T. shepardi*, and Tge = *T. genevievae.* Boxes represent the interquartile range (25th and 75th percentiles) of the data with the line inside the box representing the median. Whiskers show the most extreme value in the data which is no more than 1.5 times the interquartile range from the box. Source data are provided as a Source Data file.

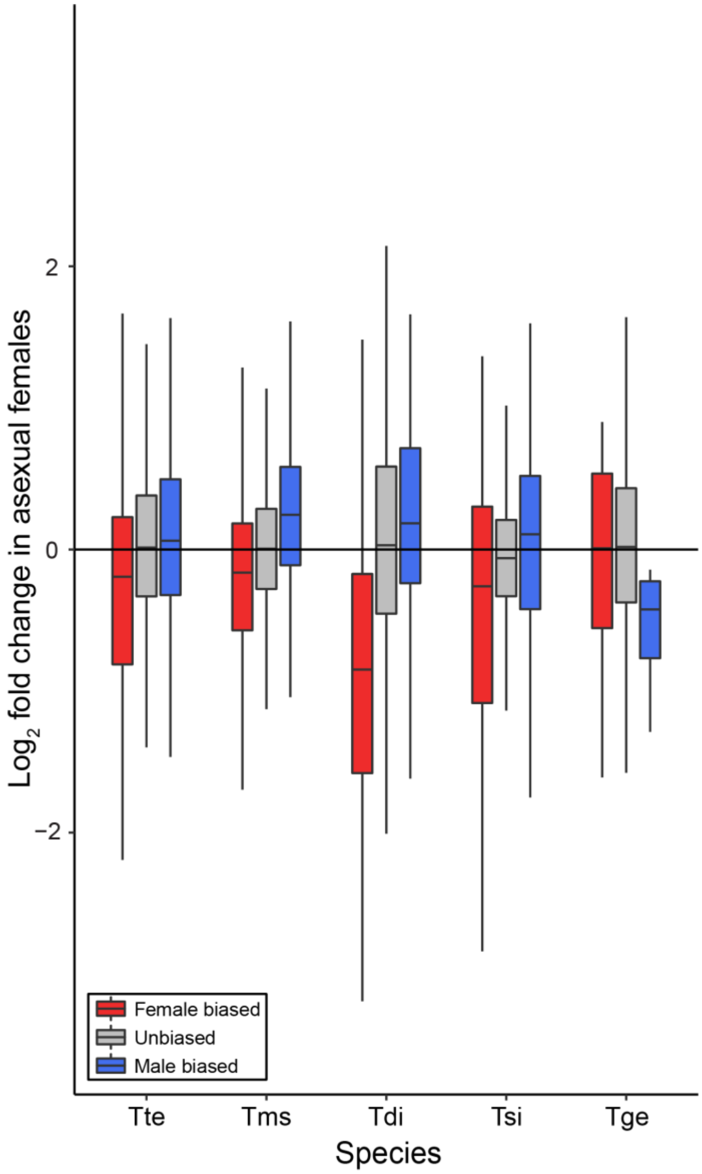

Supplementary Figure 14 | Expression shift in sex-biased genes in asexual females in whole-bodies. Sex-biased genes used were identified using both mated and virgin sexual females to independently verify sex-biased genes. Species names are abbreviated as follows: Tbi = *T. bartmani*, Tce = *T. cristinae*, Tps = *T. poppensis*, Tcm = *T. californicum*, Tpa = *T. podura*, Tte = *T. tahoe*, Tms = *T. monikensis*, Tdi = *T. douglasi*, Tsi = *T. shepardi*, and Tge = *T. genevievae.* Boxes represent the interquartile range (25th and 75th percentiles) of the data with the line inside the box representing the median. Whiskers show the most extreme value in the data which is no more than 1.5 times the interquartile range from the box. Source data are provided as a Source Data file.

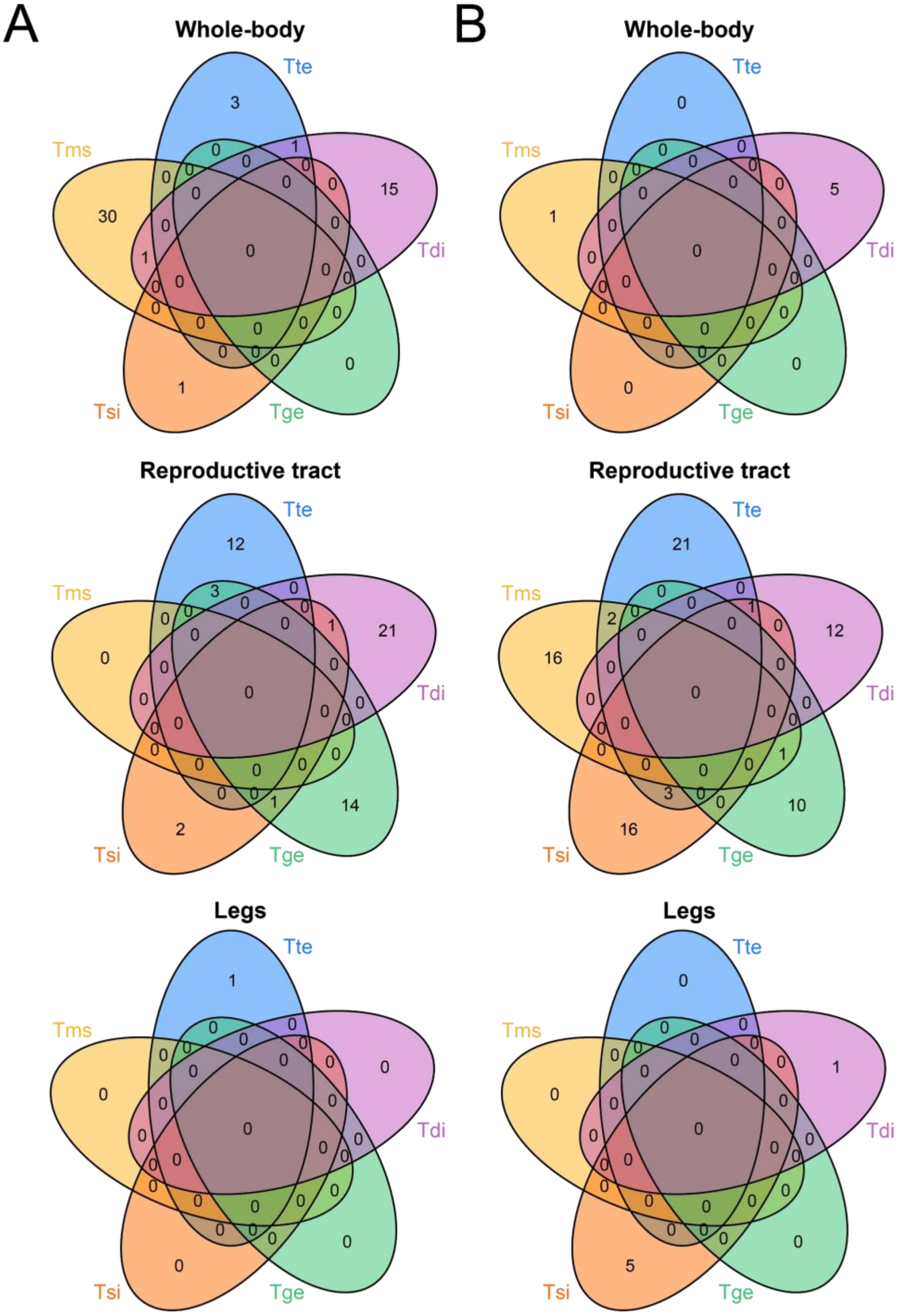

Supplementary Figure 15 | Overlap of GO-terms enriched in A) female-biased genes with decreased expression in asexual females, and in B) male-biased genes with increased expression in asexual females using NCBI's nr-arthropod GO annotation. Species names are abbreviated as follows: Tte = *T. tahoe*, Tms = *T. monikensis*, Tdi = *T. douglasi*, Tsi = *T.shepardi*, and Tge = *T. genevievae*.

Supplementary Table 1 | Numbers of sex-biased genes in each species. N FB, N MB, and N SB = number of female-, male-, or sex- biased genes respectively. SB dir = sex-bias direction, FDR = adjusted p-value for an excess of male- or female- biased genes. Species names are abbreviated as follows: Tbi = *T. bartmani*, Tce = *T. cristinae*, Tps = *T. poppensis*, Tcm = *T. californicum*, and Tpa = *T. podura*. Tissue types are abbreviated as follows: WB = whole-body, RT = reproductive tract, and LG = legs.

Species	Tissue	N FB	MB_N	SB_N	SB dir	FDR
**Tbi**	WB	529 (3.46)	625 (4.09)	1154 (7.55)	MB	**0.0083**
	RT	1788 (13.92)	2057 (16.02)	3845 (29.94)	MB	**0.0000**
	LG	241 (2)	342 (2.83)	583 (4.83)	MB	**0.0001**
**Tce**	WB	946 (6.37)	846 (5.7)	1792 (12.07)	FB	**0.0234**
	RT	1863 (15.22)	1734 (14.16)	3597 (29.38)	FB	**0.0360**
	LG	69 (0.63)	117 (1.07)	186 (1.7)	MB	**0.0010**
**Tps**	WB	697 (5.03)	479 (3.46)	1176 (8.49)	FB	**0.0000**
	RT	1656 (14.1)	2020 (17.2)	3676 (31.29)	MB	**0.0000**
	LG	131 (1.17)	219 (1.95)	350 (3.12)	MB	**0.0000**
**Tcm**	WB	168 (1.2)	121 (0.87)	289 (2.07)	FB	**0.0088**
	RT	1346 (11.82)	1497 (13.14)	2843 (24.96)	MB	**0.0083**
	LG	50 (0.45)	115 (1.03)	165 (1.48)	MB	**0.0000**
**Tpa**	WB	62 (0.45)	5 (0.04)	67 (0.48)	FB	**0.0000**
	RT	1909 (17.24)	1755 (15.85)	3664 (33.1)	FB	**0.0146**
	LG	70 (0.71)	108 (1.09)	178 (1.8)	MB	**0.0083**

Supplementary Table 2 | Common biological coefficient of variation across male and female samples for each sexual species.

Species	Whole-body	Rep. tract	Legs
T. bartmani	0.358	0.186	0.153
T. cristinae	0.324	0.415	0.294
T. poppensis	0.418	0.241	0.278
T. californicum	0.483	0.375	0.349
T. podura	0.426	0.317	0.290

Supplementary Table 3 | Wilcoxon tests for expression shifts in sex-biased genes in asexual females. ΔFB and ΔMB are the median shifts of female-biased and male-biased expression in asexual females respectively. Tissue types are abbreviated as follows: WB = whole-body, RT = reproductive tract, and LG = legs. Species names are abbreviated as follows: Tte = *T. tahoe*, Tms = *T. monikensis*, Tdi = *T. douglasi*, Tsi = *T. shepardi*; Tge = *T. genevievae.*

Sp	Tissue	ΔFB	FB FDR	ΔMB	MB FDR
Tte	WB	-0.249	**1.11×10^{-25}**	0.139	**9.72×10^{-06}**
Tms	WB	-0.178	**1.24×10^{-27}**	0.245	**9.83×10^{-39}**
Tdi	WB	-0.848	**3.50×10^{-193}**	0.749	**2.48×10^{-65}**
Tsi	WB	-0.283	**9.00×10^{-06}**	0.223	**3.48×10^{-06}**
Tge	WB	-0.184	0.0755	-0.252	0.2749
Tte	RT	-0.179	**3.50×10^{-50}**	0.223	**1.30×10^{-70}**
Tms	RT	-0.377	**1.68×10^{-110}**	0.379	**8.08×10^{-142}**
Tdi	RT	-0.071	**3.00×10^{-09}**	0.147	**4.40×10^{-33}**
Tsi	RT	-0.009	**2.14×10^{-06}**	-0.025	**0.0090**
Tge	RT	-0.252	**1.01×10^{-61}**	0.111	**0.0016**
Tte	LG	-0.421	**1.30×10^{-18}**	-0.232	**0.0016**
Tms	LG	-0.169	**0.0045**	0.051	0.3360
Tdi	LG	-0.654	**1.63×10^{-14}**	0.314	**3.84×10^{-12}**
Tsi	LG	-0.291	**6.24×10^{-05}**	-0.054	0.8031
Tge	LG	-0.503	**2.14×10^{-06}**	0.277	**0.0014**

Supplementary Table 4 | Wilcoxon tests for changes in expression in female-limited genes. SM = expression (FPKM) in males, SF = expression (FPKM) in sexual females, AF = expression (FPKM) in asexual females. N genes = Number of female-limited genes. Tissue types are abbreviated as follows: WB = whole-body, RT = reproductive tract, and LG = legs. Species names are abbreviated by the sexual species name as follows (sexual species – asexual species): Tbi = *T. bartmani - T. tahoe*; Tce = *T. cristinae - T. monikensis*; Tps = *T. poppensis - T. douglasi*; Tcm = *T. californicum - T. shepardi*; Tpa = *T. podura - T. genevievae.*

**Species**	**Tissue**	**SM exp**	**SF exp**	**AF exp**	**N genes**	**FDR**
Tbi	WB	0	9.93	11.33	39	**7.64×10^{-11}**
Tce	WB	0	7.83	5.87	50	**4.09×10^{-09}**
Tps	WB	0	16.51	6.44	50	**4.09×10^{-09}**
Tcm	WB	0	7.86	5.83	21	**2.86×10^{-06}**
Tpa	WB	0	9.16	1.85	1	1
Tbi	RT	0	13.85	10.30	33	**2.44×10^{-09}**
Tce	RT	0	20.06	9.07	29	**1.05×10^{-05}**
Tps	RT	0	11.43	4.46	13	**4.66×10^{-04}**
Tcm	RT	0	21.10	6.38	15	**1.28×10^{-04}**
Tpa	RT	0	44.34	19.62	30	**7.82×10^{-09}**
Tbi	LG	0	4.75	0.020	1	1
Tce	LG	NA	NA	NA	0	NA
Tps	LG	NA	NA	NA	0	NA
Tcm	LG	NA	NA	NA	0	NA
Tpa	LG	NA	NA	NA	0	NA

Supplementary Table 5 | Wilcoxon tests for changes in expression in male-limited genes. SM = expression (FPKM) in males, SF = expression (FPKM) in sexual females, AF = expression (FPKM) in asexual females. N genes = Number of male-limited genes. Tissue types are abbreviated as follows: WB = whole-body, RT = reproductive tract, and LG = legs. Species names are abbreviated by the sexual species name as follows (sexual species – asexual species): Tbi = *T. bartmani - T. tahoe*; Tce = *T. cristinae - T. monikensis*; Tps = *T. poppensis - T. douglasi*; Tcm = *T. californicum - T. shepardi*; Tpa = *T. podura - T. genevievae.*

**Species**	**Tissue**	**SM exp**	**SF exp**	**AF exp**	**N genes**	**FDR**
Tbi	WB	7.48	0	2.60	2	0.70
Tce	WB	NA	NA	NA	0	NA
Tps	WB	18.51	0	3.29	1	1
Tcm	WB	31.59	0	4.11	1	1
Tpa	WB	NA	NA	NA	0	NA
Tbi	RT	9.85	0	0.07	26	**1.04×10^{-07}**
Tce	RT	7.14	0	0.12	17	**3.56×10^{-05}**
Tps	RT	7.69	0	0.04	11	**1.58×10^{-03}**
Tcm	RT	14.45	0	0	5	0.47
Tpa	RT	11.03	0	0.25	11	**1.58×10^{-03}**
Tbi	LG	7.26	0	0	1	1
Tce	LG	NA	NA	NA	0	NA
Tps	LG	NA	NA	NA	0	NA
Tcm	LG	4.52	0	0	1	1
Tpa	LG	NA	NA	NA	0	NA

Supplementary Table 6 | Number of male-biased genes excluded due to low expression in asexual females. MB_excl = number of male-biased genes excluded, total_excl = total number of genes excluded, MB = number of male-biased genes in the main analysis, total = number of genes in the main analysis, fisher_OR = odd-ratio from a Fisher's exact test, fisher_FDR = FDR value from the Fisher's exact test. Tissue types are abbreviated as follows: WB = whole-body, RT = reproductive tract, and LG = legs. Species names are abbreviated by the sexual species name as follows (sexual species – asexual species): Tbi = *T. bartmani - T. tahoe*; Tce = *T. cristinae - T. monikensis*; Tps = *T. poppensis - T. douglasi*; Tcm = *T. californicum - T. shepardi*; Tpa = *T. podura - T. genevievae.*

Sp	Tiss	MB_excl	total_excl	MB	total	fisher_OR	fisher_FDR
Tbi	WB	7	119	625	15282	1.47	0.5180
Tce	WB	14	240	846	14842	1.02	0.9517
Tcm	WB	2	195	121	13959	1.19	0.7935
Tpa	WB	1	200	5	13911	13.97	0.1368
Tps	WB	3	144	479	13850	0.59	0.6161
Tbi	RT	67	225	2057	12843	2.22	**0.0000**
Tce	RT	59	241	1734	12243	1.96	**0.0001**
Tcm	RT	55	266	1497	11390	1.72	**0.0017**
Tpa	RT	97	334	1755	11070	2.17	**0.0000**
Tps	RT	59	230	2020	11747	1.66	**0.0031**
Tbi	LG	25	258	342	12077	3.68	**0.0000**
Tce	LG	3	172	117	10948	1.64	0.5951
Tcm	LG	9	206	115	11143	4.38	**0.0013**
Tpa	LG	7	651	108	9914	0.99	1.0000
Tps	LG	10	269	219	11214	1.94	0.1327

Supplementary Table 7 | Number of female-biased genes excluded due to low expression in asexual females. FB_excl = number of female-biased genes excluded, total_excl = total number of genes excluded, FB = number of female-biased genes in the main analysis, total = number of genes in the main analysis, fisher_OR = odd-ratio from a Fisher's exact test, fisher_FDR = FDR value from the Fisher's exact test. Tissue types are abbreviated as follows: WB = whole-body, RT = reproductive tract, and LG = legs. Species names are abbreviated by the sexual species name as follows (sexual species – asexual species): Tbi = *T. bartmani - T. tahoe*; Tce = *T. cristinae - T. monikensis*; Tps = *T. poppensis - T. douglasi*; Tcm = *T. californicum - T. shepardi*; Tpa = *T. podura - T. genevievae.*

Sp	Tiss	FB_excl	total_excl	FB	total	fisher_OR	fisher_FDR
Tbi	WB	1	119	529	15282	0.24	0.3279
Tce	WB	2	240	946	14842	0.12	**0.0002**
Tcm	WB	1	195	168	13959	0.42	0.9163
Tpa	WB	1	200	62	13911	1.12	0.8099
Tps	WB	1	144	697	13850	0.13	**0.0269**
Tbi	RT	12	225	1788	12843	0.35	**0.0002**
Tce	RT	7	241	1863	12243	0.17	**0.0000**
Tcm	RT	6	266	1346	11390	0.17	**0.0000**
Tpa	RT	13	334	1909	11070	0.19	**0.0000**
Tps	RT	27	230	1656	11747	0.81	0.5083
Tbi	LG	9	258	241	12077	1.78	0.2374
Tce	LG	1	172	69	10948	0.92	1.0000
Tcm	LG	0	206	50	11143	0.00	1.0000
Tpa	LG	1	651	70	9914	0.22	0.2455
Tps	LG	3	269	131	11214	0.95	1.0000

Supplementary Table 8 | Number of male-biased genes excluded due to low expression in asexual females when using the sexual reference. MB_excl = number of male-biased genes excluded, total_excl = total number of genes excluded, MB = number of male-biased genes in the main analysis, total = number of genes in the main analysis, fisher_OR = odd-ratio from a Fisher's exact test, fisher_FDR = FDR value from the Fisher's exact test. Tissue types are abbreviated as follows: WB = whole-body, RT = reproductive tract, and LG = legs. Species names are abbreviated by the sexual species name as follows (sexual species – asexual species): Tbi = *T. bartmani - T. tahoe*; Tce = *T. cristinae - T. monikensis*; Tps = *T. poppensis - T. douglasi*; Tcm = *T. californicum - T. shepardi*; Tpa = *T. podura - T. genevievae.*

sp	Tiss	MB_excl	total_excl	MB_kept	total_kept	MB_fisher_OR	MB_fisher_FDR
Tbi	WB	93	758	969	19426	2.66	**0.0000**
Tce	WB	110	1069	1116	19757	1.92	**0.0000**
Tcm	WB	21	704	160	17661	3.36	**0.0000**
Tpa	WB	1	1383	6	22296	2.69	0.3684
Tps	WB	31	872	560	17862	1.14	0.4865
Tbi	GN	190	602	2634	16105	2.36	**0.0000**
Tce	GN	118	534	2292	16019	1.70	**0.0000**
Tcm	GN	108	500	1843	14412	1.88	**0.0000**
Tpa	GN	215	810	2644	16838	1.94	**0.0000**
Tps	GN	137	583	2584	15242	1.50	**0.0001**
Tbi	LG	75	654	474	15161	4.01	**0.0000**
Tce	LG	9	512	171	14761	1.53	0.2401
Tcm	LG	19	584	168	13733	2.71	**0.0003**
Tpa	LG	28	1602	164	15022	1.61	**0.0325**
Tps	LG	30	683	324	14497	2.01	**0.0013**

Supplementary Table 9 | Number of female-biased genes excluded due to low expression in asexual females when using the sexual reference. FB_excl = number of female-biased genes excluded, total_excl = total number of genes excluded, FB = number of female-biased genes in the main analysis, total = number of genes in the main analysis, fisher_OR = odd-ratio from a Fisher's exact test, fisher_FDR = FDR value from the Fisher's exact test. Tissue types are abbreviated as follows: WB = whole-body, RT = reproductive tract, and LG = legs. Species names are abbreviated by the sexual species name as follows (sexual species – asexual species): Tbi = *T. bartmani - T. tahoe*; Tce = *T. cristinae - T. monikensis*; Tps = *T. poppensis - T. douglasi*; Tcm = *T. californicum - T. shepardi*; Tpa = *T. podura - T. genevievae.*

Sp	Tiss	FB_excl	total_excl	FB_kept	total_kept	FB_fisher_OR	FB_fisher_FDR
Tbi	WB	8	758	582	19426	0.35	**0.0021**
Tce	WB	15	1069	1209	19757	0.22	**0.0000**
Tcm	WB	3	704	186	17661	0.40	0.1346
Tpa	WB	0	1383	73	22296	0.00	**0.0366**
Tps	WB	24	872	772	17862	0.63	**0.0373**
Tbi	GN	30	602	2182	16105	0.33	**0.0000**
Tce	GN	19	534	2282	16019	0.22	**0.0000**
Tcm	GN	14	500	1631	14412	0.23	**0.0000**
Tpa	GN	44	810	2765	16838	0.29	**0.0000**
Tps	GN	54	583	2106	15242	0.64	**0.0029**
Tbi	LG	22	654	289	15161	1.79	**0.0256**
Tce	LG	0	512	91	14761	0.00	0.0969
Tcm	LG	1	584	59	13733	0.40	0.5197
Tpa	LG	4	1602	95	15022	0.39	0.0809
Tps	LG	13	683	169	14497	1.64	0.1167

Supplementary Table 10 | Number of male-biased genes excluded due to low expression in asexual females when using the asexual reference. MB_excl = number of male-biased genes excluded, total_excl = total number of genes excluded, MB = number of male-biased genes in the main analysis, total = number of genes in the main analysis, fisher_OR = odd-ratio from a Fisher's exact test, fisher_FDR = FDR value from the Fisher's exact test. Tissue types are abbreviated as follows: WB = whole-body, RT = reproductive tract, and LG = legs. Species names are abbreviated by the sexual species name as follows (sexual species – asexual species): Tbi = *T. bartmani - T. tahoe*; Tce = *T. cristinae - T. monikensis*; Tps = *T. poppensis - T. douglasi*; Tcm = *T. californicum - T. shepardi*; Tpa = *T. podura - T. genevievae.*

sp	Tiss	MB_excl	total_excl	MB_kept	total_kept	MB_fisher_OR	MB_fisher_FDR
Tbi	WB	13	352	886	23653	0.99	1.0000
Tce	WB	37	618	1193	24292	1.23	0.3553
Tcm	WB	5	647	105	22607	1.67	0.3553
Tpa	WB	1	655	10	22943	3.51	0.3632
Tps	WB	9	531	493	21428	0.73	0.4936
Tbi	GN	147	604	3129	20237	1.76	0.0000
Tce	GN	86	514	2610	20487	1.38	0.0196
Tcm	GN	182	718	2300	19535	2.54	0.0000
Tpa	GN	165	752	2794	19073	1.64	0.0000
Tps	GN	144	515	3168	19169	1.96	0.0000
Tbi	LG	48	630	467	17876	3.07	0.0000
Tce	LG	7	373	118	17023	2.74	0.0341
Tcm	LG	14	629	138	16748	2.74	0.0032
Tpa	LG	10	835	145	16234	1.34	0.4009
Tps	LG	14	585	322	17455	1.30	0.4009

Supplementary Table 11 | Number of female-biased genes excluded due to low expression in asexual females when using the asexual reference. FB_excl = number of female-biased genes excluded, total_excl = total number of genes excluded, FB = number of female-biased genes in the main analysis, total = number of genes in the main analysis, fisher_OR = odd-ratio from a Fisher's exact test, fisher_FDR = FDR value from the Fisher's exact test. Tissue types are abbreviated as follows: WB = whole-body, RT = reproductive tract, and LG = legs. Species names are abbreviated by the sexual species name as follows (sexual species – asexual species): Tbi = *T. bartmani - T. tahoe*; Tce = *T. cristinae - T. monikensis*; Tps = *T. poppensis - T. douglasi*; Tcm = *T. californicum - T. shepardi*; Tpa = *T. podura - T. genevievae.*

sp	Tiss	FB_excl	total_excl	FB_kept	total_kept	FB_fisher_OR	FB_fisher_FDR
Tbi	WB	1	352	620	23653	0.11	0.0041
Tce	WB	3	618	1416	24292	0.08	0.0000
Tcm	WB	0	647	175	22607	0.00	0.0321
Tpa	WB	0	655	92	22943	0.00	0.2557
Tps	WB	11	531	798	21428	0.55	0.0697
Tbi	GN	21	604	2904	20237	0.22	0.0000
Tce	GN	13	514	2744	20487	0.17	0.0000
Tcm	GN	5	718	2019	19535	0.06	0.0000
Tpa	GN	38	752	3039	19073	0.28	0.0000
Tps	GN	35	515	2623	19169	0.46	0.0000
Tbi	LG	21	630	354	17876	1.71	0.0484
Tce	LG	0	373	87	17023	0.00	0.3349
Tcm	LG	0	629	46	16748	0.00	0.4786
Tpa	LG	3	835	99	16234	0.59	0.5257
Tps	LG	7	585	204	17455	1.02	0.8452

Supplementary Table 12 | Numbers of sex-biased genes in each species when using the 10-species orthologs. N FB, N MB, and N SB = number of female-, male-, or sex- biased genes respectively. SB dir = sex-bias direction, FDR = adjusted p-value for an excess of male- or female- biased genes. Species names are abbreviated as follows: Tbi = *T. bartmani*, Tce = *T. cristinae*, Tps = *T. poppensis*, Tcm = *T. californicum*, and Tpa = *T. podura*. Tissue types are abbreviated as follows: WB = whole-body, RT = reproductive tract, and LG = legs.

Species	Tissue	N FB	N MB	N SB	SB dir	FDR
Tbi	WB	124 (4.15)	158 (5.28)	282 (9.43)	MB	0.0883
	RT	371 (13.20)	520 (18.51)	891 (31.71)	MB	**0.0000**
	LG	73 (2.60)	84 (2.99)	157 (5.59)	MB	0.4343
Tce	WB	188 (6.28)	187 (6.24)	375 (12.52)	FB	0.9834
	RT	378 (13.27)	429 (15.06)	807 (28.34)	MB	0.1263
	LG	26 (0.93)	38 (1.35)	64 (2.28)	MB	0.1979
Tps	WB	174 (5.82)	174 (5.82)	348 (11.63)	NB	1.0000
	RT	513 (18.16)	558 (19.75)	1071 (37.91)	MB	0.2333
	LG	31 (1.10)	49 (1.74)	80 (2.85)	MB	0.0883
Tcm	WB	57 (1.91)	77 (2.58)	134 (4.49)	MB	0.1401
	RT	360 (12.75)	424 (15.02)	784 (27.77)	MB	0.0636
	LG	18 (0.64)	31 (1.11)	49 (1.75)	MB	0.1151
Tpa	WB	29 (0.97)	14 (0.47)	43 (1.44)	FB	0.0636
	RT	545 (19.40)	516 (18.36)	1061 (37.76)	FB	0.4343
	LG	23 (0.82)	43 (1.54)	66 (2.36)	MB	0.0591

Supplementary Table 13 | Wilcoxon tests for expression shifts in sex-biased genes in asexual females when using 1-to-1 orthologs. ΔFB and ΔMB are the median shifts of female-biased and male-biased expression in asexual females respectively. Tte = *T. tahoe*, Tms = *T. monikensis*, Tdi = *T. douglasi*, Tsi = *T. shepardi*; Tge = *T. genevievae.* Tissue types are abbreviated as follows: WB = whole-body, RT = reproductive tract, and LG = legs.

**Sp**	**Tissue**	**ΔFB**	**FB FDR**	**ΔMB**	**MB FDR**
Tte	WB	-0.217	**9.52×10^{-05}**	0.035	0.5223
Tms	WB	-0.224	**1.48×10^{-11}**	0.214	**1.37×10^{-13}**
Tdi	WB	-0.627	**1.06×10^{-36}**	0.318	**2.13×10^{-14}**
Tsi	WB	0.175	0.2603	-0.156	0.2425
Tge	WB	-0.064	0.5583	-0.049	0.5570
Tte	RT	-0.171	**1.48×10^{-11}**	0.161	**3.39×10^{-12}**
Tms	RT	-0.493	**1.22×10^{-44}**	0.453	**1.12×10^{-30}**
Tdi	RT	-0.103	**3.30×10^{-04}**	0.152	**2.03×10^{-15}**
Tsi	RT	0.014	0.1189	-0.023	0.0607
Tge	RT	-0.191	**8.22×10^{-12}**	0.145	**0.0037**
Tte	LG	-0.507	**2.87×10^{-05}**	-0.134	0.4794
Tms	LG	-0.244	**0.0232**	0.130	**0.0484**
Tdi	LG	-0.827	**3.88×10^{-04}**	0.506	**9.30×10^{-06}**
Tsi	LG	-0.073	0.4751	-0.103	0.5408
Tge	LG	-0.464	**0.0069**	0.446	**0.0457**

Supplementary Table 14 | FDR and gradients for the relationship between the number of species a gene is sex-biased in and the shift in expression in asexual females from a GLM (GLM_FDR) and a GLMM (GLMM_FDR) where gene ID was included as a random effect. Sex-bias = Female-biased (FB) or male-biased (MB) genes. Tissue types are abbreviated as follows: WB = whole-body, RT = reproductive tract, and LG = legs.

Sex-bias	Tissue	Gradient	GLM_FDR	GLMM_FDR
FB	WB	0.09099236	0.00252778	0.02017974
FB	RT	0.07658775	9.09E-09	3.53E-08
FB	LG	0.14406285	0.01665674	0.02218378
MB	WB	-0.0942748	0.0012744	0.0012744
MB	RT	-0.0440438	0.00223275	0.01255613
MB	LG	-0.1891408	0.00631202	0.02218378

Supplementary Table 15 | The effects of reproductive mode and sex-bias on dN/dS when modelled using a glmm approach. LRT_bi = log-likelihood ratio test statistic for the binomial model, p_bi = p-value for the binomial model, LRT_nz = log-likelihood ratio test statistic for the gamma model, p_nz = p-value for the gamma model. Tissue types are abbreviated as follows: WB = whole-body, RT = reproductive tract, and LG = legs.

Tissue	term	LRT_bi	p_bi	LRT_nz	p_nz
WB	rep mode	268.221	< 2e-16	62.858	2.22E-15
WB	sex bias	7.046	0.02951	6.549	0.03783
WB	rep mode:sex bias	0.69475	0.7065	4.8613	0.08798
WB	OG_name	436.736047	< 2e-16	541.579247	< 2e-16
RT	rep mode	266.388	< 2e-16	75.116	< 2e-16
RT	sex bias	6.787	0.03359	0.94	0.6251
RT	rep mode:sex bias	3.5051	0.1733	0.0082594	0.9959
RT	OG_name	424.217296	< 2e-16	506.536595	< 2e-16
LG	rep mode	261.564	< 2e-16	71.029	< 2e-16
LG	sex bias	11.477	0.003219	2.687	0.2609
LG	rep mode:sex bias	5.3507	0.06888	1.3921	0.4985
LG	OG_name	392.431522	< 2e-16	498.694099	< 2e-16

Supplementary Table 16 | The effects of reproductive mode and sex-bias on dN/dS when modelled using a glmm approach, excluding female-biased genes. LRT_bi = log-likelihood ratio test statistic for the binomial model, p_bi = p-value for the binomial model, LRT_nz = log-likelihood ratio test statistic for the gamma model, p_nz = p-value for the gamma model. Tissue types are abbreviated as follows: WB = whole-body, RT = reproductive tract, and LG = legs.

Tissue	Term	LRT_bi	p_bi	LRT_nz	p_nz
WB	rep mode	264.049	< 2e-16	62.774	2.32E-15
WB	sex bias	1.103	0.2935	3.762	0.05242
WB	rep mode:sex bias	0.0180558	0.8931	3.1135	0.07765
WB	OG_name	405.501086	< 2e-16	540.151184	< 2e-16
RT	rep mode	242.251	< 2e-16	62.571	2.57E-15
RT	sex bias	6.051	0.0139	0.107	0.7434
RT	rep mode:sex bias	1.4708	0.2252	0.019517	0.8889
RT	OG_name	333.263803	< 2e-16	432.166011	< 2e-16
LG	rep mode	264.966	< 2e-16	71.214	< 2e-16
LG	sex bias	9.469	0.00209	0.391	0.5317
LG	rep mode:sex bias	1.5662	0.2108	0.17648	0.6744
LG	OG_name	386.730801	< 2e-16	491.822732	< 2e-16

Supplementary Table 17 | The effects of reproductive mode and sex-bias on dN/dS when modelled using a glmm approach, excluding male-biased genes. LRT_bi = log-likelihood ratio test statistic for the binomial model, p_bi = p-value for the binomial model, LRT_nz = log-likelihood ratio test statistic for the gamma model, p_nz = p-value for the gamma model. Tissue types are abbreviated as follows: WB = whole-body, RT = reproductive tract, and LG = legs.

Tissue	Term	LRT_bi	p_bi	LRT_nz	p_nz
WB	rep mode	261.685	< 2e-16	64.44	9.95E-16
WB	sex bias	6.315	0.01197	3.337	0.06772
WB	rep mode:sex bias	0.66007	0.4165	1.9975	0.1576
WB	OG_name	412.992912	< 2e-16	520.666943	< 2e-16
RT	rep mode	222.618	< 2e-16	57.96	2.68E-14
RT	sex bias	0.382	0.54	0.982	0.3216
RT	rep mode:sex bias	2.6293	0.10	0.00258293	0.9595
RT	OG_name	291.599002	< 2e-16	386.117859	< 2e-16
LG	rep mode	252.98	< 2e-16	67.225	2.42E-16
LG	sex bias	1.675	0.1956	2.32	0.1274
LG	rep mode:sex bias	3.7015	0.05436	1.214	0.2705
LG	OG_name	380.138803	< 2e-16	489.202805	< 2e-16

Supplementary Table 18 | Numbers of sex-biased genes in each species when using only an FDR threshold. N FB, N MB, and N SB = number of female-, male-, or sex- biased genes respectively. SB dir = sex-bias direction, FDR = adjusted p-value for an excess of male- or female- biased genes. Species names are abbreviated as follows: Tbi = *T. bartmani*, Tce = *T. cristinae*, Tps = *T. poppensis*, Tcm = *T. californicum*, and Tpa = *T. podura*. Tissue types are abbreviated as follows: WB = whole-body, RT = reproductive tract, and LG = legs.

Species	Tissue	N FB	MB_N	SB_N	SB dir	FDR
Tbi	WB	562 (3.68)	643 (4.21)	1205 (7.89)	MB	**0.0245**
	RT	3830 (29.82)	3735 (29.08)	7565 (58.9)	FB	0.2892
	LG	739 (6.12)	842 (6.97)	1581 (13.09)	MB	**0.0132**
Tce	WB	1235 (8.32)	1024 (6.9)	2259 (15.22)	FB	**0.0000**
	RT	1987 (16.23)	1849 (15.1)	3836 (31.33)	FB	**0.0314**
	LG	83 (0.76)	133 (1.21)	216 (1.97)	MB	**0.0015**
Tps	WB	697 (5.03)	479 (3.46)	1176 (8.49)	FB	**0.0000**
	RT	3142 (26.75)	3209 (27.32)	6351 (54.06)	MB	0.4108
	LG	159 (1.42)	246 (2.19)	405 (3.61)	MB	**0.0000**
Tcm	WB	168 (1.2)	121 (0.87)	289 (2.07)	FB	**0.0088**
	RT	1595 (14)	1701 (14.93)	3296 (28.94)	MB	**0.0701**
	LG	50 (0.45)	116 (1.04)	166 (1.49)	MB	**0.0000**
Tpa	WB	62 (0.45)	5 (0.04)	67 (0.48)	FB	**0.0000**
	RT	2495 (22.54)	2307 (20.84)	4802 (43.38)	FB	**0.0098**
	LG	76 (0.77)	115 (1.16)	191 (1.93)	MB	**0.0083**

Supplementary Table 19 | Wilcox tests for expression shift in sex-biased genes in asexual females for when sex-biased genes are classed using only an FDR threshold. Tte = *T. tahoe*, Tms = *T. monikensis*, Tdi = *T. douglasi*, Tsi = *T. shepardi*; Tge = *T. genevievae.* Tissue types are abbreviated as follows: WB = whole-body, RT = reproductive tract, and LG = legs.

**Sp**	**Tissue**	**ΔFB**	**FB FDR**	**ΔMB**	**MB FDR**
Tte	WB	-0.255	**7.29×10^{-28}**	0.136	**1.80×10^{-05}**
Tms	WB	-0.164	**1.33×10^{-38}**	0.211	**6.82×10^{-34}**
Tdi	WB	-0.848	**3.50×10^{-193}**	0.749	**3.73×10^{-65}**
Tsi	WB	-0.283	**9.69×10^{-06}**	0.223	**3.04×10^{-06}**
Tge	WB	-0.184	0.0755	-0.252	0.2587
Tte	RT	-0.126	**3.73×10^{-51}**	0.162	**4.26×10^{-48}**
Tms	RT	-0.368	**4.92×10^{-113}**	0.351	**3.56×10^{-134}**
Tdi	RT	-0.063	**1.07×10^{-17}**	0.087	**1.55×10^{-11}**
Tsi	RT	0.009	**3.76×10^{-04}**	-0.008	0.0541
Tge	RT	-0.199	**2.16×10^{-61}**	0.087	0.4055
Tte	LG	-0.252	**1.55×10^{-37}**	-0.087	**2.03×10^{-06}**
Tms	LG	-0.175	**0.0024**	0.051	0.1464
Tdi	LG	-0.590	**1.44×10^{-17}**	0.314	**1.41×10^{-12}**
Tsi	LG	-0.291	**6.68×10^{-05}**	-0.070	0.7539
Tge	LG	-0.503	**4.15×10^{-07}**	0.274	**9.61×10^{-04}**

Supplementary References

1. Bast, J. *et al.* Consequences of asexuality in natural populations: insights from stick insects. *Mol. Biol. Evol.* **35**, 1668–1677 (2018).
