# Supplementary material for: Sex-biased gene expression is repeatedly masculinized in asexual females
Source: Nat Commun. 2019 Oct 11;10:4638. doi: 10.1038/s41467-019-12659-8 (PMC6789136; doi:10.1038/s41467-019-12659-8)
Supplement: Supplementary file 3 — Description of Additional Supplementary Files [file 41467_2019_12659_MOESM3_ESM.pdf]

Description of Additional Supplementary Files

File Name: Supplementary Data 1
Description: GO terms enriched in sex-biased genes

File Name: Supplementary Data 2
Description: Overlap of enriched GO terms in sex-biased genes between species

File Name: Supplementary Data 3
Description: Overlap male-biased and female-biased genes between species

File Name: Supplementary Data 4
Description: GO terms enriched in female-biased genes that decrease in expression in asexual females

File Name: Supplementary Data 5
Description: GO terms enriched in male-biased genes that increase in expression in asexual females

File Name: Supplementary Data 6
Description: GO terms enriched in female-biased genes that increase in expression in asexual females

File Name: Supplementary Data 7
Description: GO terms enriched in male-biased genes that decrease in expression in asexual females

File Name: Supplementary Data 8
Description: Sample information
